# Intimate partner problems and suicide: are we missing the violence?

**DOI:** 10.5249/jivr.v11i1.997

**Published:** 2019-01

**Authors:** Sabrina Brown, Jacqueline Seals

**Affiliations:** ^*a*^Department of Epidemiology, University of Kentucky, Kentucky Violent Death Reporting System, Kentucky, USA.

**Keywords:** Suicide, Domestic, Violence, Abuse

## Abstract

**Background:**

Suicide consistently ranks in the top ten causes of death nationally. The purpose of this study was to develop a novel coding scheme to determine what percentage of suicide cases from 2005-2015 in Kentucky involved violence when intimate partner problems were identified. Currently, researchers using the national dataset, containing these data, only have the option to identify in-timate partner problems unless each case is reviewed individually.

**Methods:**

Data from the Kentucky Violent Death Reporting System from 2005-2015 were used to create a subset of cases where intimate partner problems were identified and qualitative and quantita-tive analysis of the death scene investigation incident narratives was conducted to identify cases where intimate partner violence also contributed to the suicide.

**Results:**

Intimate partner problems were identified in 1,327 (26%) of all suicide cases where circumstances were known and intimate partner violence in 575 (43%) cases identified as having intimate partner problems. There was an argument or fight in 30% of cases where intimate partner problems were identified and most were immediately followed by the suicide.

**Conclusions:**

We did find supporting evidence of our hypothesis that there is a great deal of underlying and outright violence in intimate relationships, which is exacerbating the risk of suicide. This detailed coding schema guided abstractors to better identify intimate partner violence in suicides, which could be easily replicated.

## Introduction

Suicide consistently ranks in the top ten causes of death nationally.^1^ In 2014 approximately 42,826 people died by suicide.^1^ Suicide numbers have continued to increase in the United States with a 15.26% increase since 1999.^1^ The Commonwealth of Kentucky has exceeded national suicide rates every year from 2005-2012; in 2013 Kentucky’s rate was 14.8 and the nation 13.1.^1-6^ Because Kentucky has remained well above the nation, and suicides continue to increase, it is critical that studies like this continue in order to understand the problem. With this understanding prevention efforts can provide a more targeted approach and novel strategies implemented. Intra-articular injection of drugs for treatment of inflammatory and non-inflammatory joint disease can result in septic arthritis. Yet, there is no report with respect to injection of petrol as a cause of septic arthritis in the literature. In this study we report the first case of petrol- induced knee septic arthritis. 

Limitations in reporting and availability of suicide data hinder prevention efforts.^7,8^ Suicide results from a complex mix of social risk factors, population characteristics, interdependencies (i.e. co-occurring conditions such as mental illness and substance abuse), and multilevel causality. ^2-7,9-13^ In their seminal 2004 article, Knox, Cornwell and Caine stated, "social determinants of suicide are likely to contribute as much as, if not more than, individual risk factors, but they have been poorly studied to date.^10^ " Almost ten years later this remains the case. Suicide prevention must be identified as a public health priority.^14^

The capacity for prediction and prevention lie in documentation and evaluation of past mortality trends.^8^ In turn, suicide prevention efforts will benefit from identifying potential windows and foci for intervention. Risk factors for suicide have been studied extensively on an individual level. Risk factors identified consistently in the literature for intimate partner problems (IPP), intimate partner violence (IPV), violence or conflict were similar to what we identified in the incident narratives.^2-7,9,11,13-15^

Strong associations have been found between suicide and mental health issues, substance abuse, social isolation, trauma, violence, and IPPs.^9,11,16^ Depression is one of the most prevalent mental health problems in regard to both IPV and suicide; the combination of Post Traumatic Stress Disorder (PTSD) and depression can lead to negative health outcomes, not adhering to medical treatment and damaging coping behaviors.^17,18^ Violent acts are most often perpetrated by intimate partners and evolve out of relationship dissatisfaction.^19^ There is ample evidence of associations between IPV, mental illness and substance abuse; specifically, one study finds that over half of women seen at a Level 1 rural trauma center experienced lifetime IPV and had significantly high associations with mental illness, illicit substance abuse, alcohol abuse, other risk factors for injury, and among those experiencing IPV within the last year, firearm ownership by a partner.^20^ Another study tested risk factors for lethality in survivors of domestic violence; strangulation occurred in 38% of IPV cases with a significant risk for lethality.^21^ A longitudinal study examining depression and PTSD symptoms showed that when there was harm to the couple’s children the mother’s depression and PTSD symptoms increased.^22^ Less severe and often less detectable forms of abuse, such as nonviolent coersive control, verbal and psychological abuse and neglect also warrant study and screening as these cases can escalate during times of acute stresses, such as separation or divorce.^19,23^ Between 40-80% of couples entering into divorce proceedings have experienced violence and abuse within their relationship creating a power imbalance and an unfair negotiating/mediation environment, all leading to a higher risk of violence.^24,25^ Nielsen and colleagues explored severity and frequency of violence by comparing variations within situational couple violence, coercive controlling violent couples and no violence/no control couples. They found that situational couple violence was frequent and severe, resembled coercive controlling violence with no difference in mental health symptoms between groups. They attributed this to the stresses of divorce.^26^ Divorce and separation may be particularly salient risk factors for suicide in men.^27^ Marital separation may quadruple the risk of suicide, especially in younger men.^28^ There is also growing evidence that loss of custody of a child is a precipitating factor for suicide.^29^

The prevalence of smokers experiencing IPV was two to three times higher than women not experiencing IPV nationally; the co-occurring conditions of those that smoke, psychiatric symptom severity and maladaptive behaviors fared worse than those that didn’t.^30^ Co-occurring conditions can produce a particularity high risk milieu for violence. 

IPPs have been linked to increased risk of suicide. Thompson, Kaslow and Kingree contributed meaningfully to the literature when they studied cumulative risk to see if more risk factors in African American women, who had recently experienced IPV, would increase likelihood of attempting suicide. They found that women with four to five risk factors were 107 times more likely to attempt suicide than those women with no risk factors. ^31^ Ortega and Karch suggest a woman's vulnerability to suicide increases when growth is blocked or distorted in relationships, social connectedness decreases, and social isolation increases.^13^ Vulnerable times in a woman’s life can also exacerbate the consequences of IPV; one study found an association between postpartum depression and IPV.^32^ Conversely, positive coping mechanisms such as spiritual beliefs and religious practices have been found to improve mental health in suicidal survivors of IPV.^33,34^ The existential nature of religious belief; that there is vastness beyond one’s own individual humanity, lowered levels of feelings of hopelessness in survivors of IPV.^33,34^

IPP was identified as a precipitating circumstance in 30% of all suicide cases in the National Violent Death Reporting System (NVDRS) from 2005-2010 NVDRS.^2-6^ The importance of healthy relationships, free of IPP and IPV, throughout the lifespan for males and females has become a focus for violence prevention efforts.^35^ While there is extensive literature evaluating IPV and homicide,^28^ research focusing on IPV and suicide outcomes is lacking. Relationship problems, mistrust, jealousy, discord, prior physical, sexual, psychological, emotional abuse or violence might be missed, or not considered relevant to the investigation. 

The purpose of this analysis is to determine the proportion of suicides where IPP and IPV were reported as circumstances related to suicides. The only surveillance system that currently captures this circumstantial information is the NVDRS. Abstractors, who enter data from death scene investigators, are limited to identifying and coding relationship circumstances as IPPs even if there is also violence. The only way to determine if there was also violence is to read the narratives on death investigators’ reports. Information about IPV is lost if all of the narratives are not read and coded uniformly. This understanding is important to the public’s health for three reasons: 1) suicide is increasing, 2) IPPs have been linked to an increased risk for suicide and 3) distinguishing between suicides by IPV perpetrators following assaults on their partners and suicides by people experiencing IPPs is important from both an etiological and a prevention strategy perspective. Our hypothesis is that qualitative and quantitative analysis of the death scene narratives of suicides in Kentucky will reveal that there is a great deal of underlying and outright IPV precipitating suicides that are identified as only having IPPs, in materials generated using NVDRS data, which is exacerbating the risk of suicide. We will use Thomas Joiner’s interpersonal-psychological theory of suicidal behavior as a framework to present and interpret findings.

No prior method of identifying type and patterns of violence in IPP related suicide has been published and this has left a void in IPV research. This study aims to identify suicide cases with IPV, using a novel approach, to determine if there is a significant number of suicide cases with IPV that have only been identified as IPP within the NVDRS dataset.

## Methods 

***Data Source***

Data for the state of Kentucky from the National Violent Death Reporting System (NVDRS) were used for this report. NVDRS is an active state-based surveillance system that captures information from death certificates and death scene investigation reports from coroners, medical examiners, law enforcement, toxicology and forensic reports.^36^ The NVDRS has been described in further detail elsewhere. ^37,38^ The information collected includes detailed circumstance information such as a history of IPPs and/or IPV, information regarding manner of death, and the victim-suspect relationship. The Kentucky Violent Death Reporting System (KVDRS) collects information as part of NVDRS; data for this study includes all cases occurring in Kentucky, regardless of state residency.^15^

Cases were first confirmed to be suicides using the International Classification of Diseases 10th Revision (ICD-10) codes meeting CDC's definition of suicide and the manner of death.^39^ Cases were then divided into two groups; one set where the abstractor coded IPP as a precipitating circumstance to the suicide and cases where there were no IPP circumstances noted. Intimate partners were defined as: current or former spouses (including common-law spouses), non-marital dating partners (including same sex partners) boyfriend/girlfriend, sexual partners, or having a child in common regardless of marital or dating status. Intimate partner status is not dependent on cohabitation or sexual activity.^40^

IPP was defined as problems the victim experienced at the time of the incident with a current or former intimate partner, including a divorce, break-up, argument, jealousy, conflict or discord.^37^ IPV was defined as physical violence, sexual violence, current or prior threat of physical or sexual violence, and/or psychological and emotional abuse including coercive tactics with a history of prior physical or sexual violence by a current or former intimate partner.^40^

Between 2009 and 2010 data abstractors were able to select IPV as well as IPP as circumstances for suicides; 2005-2008 and 2011-2015 abstractors were restricted to only choosing IPP as a circumstance for suicide and IPV as a circumstance for homicide deaths. An open text field, called the incident narrative, gives investigators the option of recording the circumstances in their own words. Narratives from Kentucky's Coroner Investigation Report (CIR) were most often available as coroners in Kentucky investigate the cause and manner of death defined as coroners' cases by Kentucky Revised Statute (KRS) 72.025.^41^ Narratives were also evaluated, when available, from local law enforcement (LLE) reports; LLE and/or the Kentucky State Police also investigate suicides.

Each suicide case incident narrative was assessed to confirm the presence of IPP and IPV and to identify trends and patterns of terminology describing IPP and IPV. A coding scheme was created to identify when there was IPP, if there was IPV and what type of violence precipitated a suicide. Since the incident narrative is the only way to capture IPV for all years of data, cases without narratives (n=1,979) were excluded from all analyses.

***Data Analysis***

Analysis is based on a study by Holland and colleagues in which the first author participated and where coroner/medical examiner (CME) and LLE NVDRS narratives were analyzed qualitatively.^29^ Content analysis occurred in three steps: 1) development of a coding structure; 2) iterative codification, where the coding structure was revised through a multi-step method involving several narrative reviews (this step allowed for the generation of a code book, see Table 1); and 3) thematic analysis and interpretations. Additionally, the identified themes were reviewed and evaluated by three expert scholars in the field of injury prevention and epidemiology. This coding scheme allows single or multi-state analysis of IPP and IPV using consistent variables for comparison.^29,42,43^

**Table 1:** Incident narrative coded variables and definitions for Intimate Partner Problem (IPP) and Intimate Partner Violence (IPV).

Themes	Definition
**Intimate Partner Problem (IPP) **	
Divorce	Investigator report (IR) used the term "divorce"
Marital Problems	IR used the term "marital problem/s"
	Incident narrative indicated marital problem without specific wording
Problem	IR used the term "relationship problem, domestic problem, break-up, or rejection"
	Incident narrative indicated relationship problem without specific wording
Separation/Left	IR used the term "separation or left"
Intent to Leave	IR used the term "intent to leave or leaving"
Custody Problems (Child)	IR used the term "custody" or wording describing custody of children
	Incident narrative indicated child visitation was being withheld
Upset/Distraught	IR used the term "upset or distraught"
Estranged	IR used the term "estranged"
Infidelity	IR used the term "infidelity, flirting" or an IP dating others, includes “suspicion” and “suspected”
IPP not specified	Incident narrative indicated unspecified IPP or without specific wording for other IPP variables.
**Intimate Partner Violence (IPV) **	
H/S	Homicide/Suicide combination
Violence	IR used the term "violence" or “abusive”
Domestic Dispute Domestic Argument	IR used the term "domestic dispute or domestic argument"
Restraining Order	IR used the term "restraining order" any type: EPO, DVO, TRO, or TPO
Threatened Suicide	IR used the term "threatened suicide;” does not include “previous attempts, or suicidal thoughts"
Argument/Fight/Altercation	IR used the term "argument, dispute, fight, or altercation"
Assault of Another Person	IR used the term "assault, attack, abuse (not in reference to alcohol or drugs), sexual as-sault/molestation,
	IPV, stalked, harassed, threaten (to kill or harm, not suicide threat), beaten, shot, stabbed" or where non-fatal violence was against another person (not H/S)
IPV Not Specified	Incident narrative indicated unspecified IPV or without specific wording for other IPP variables.

In the second phase, incident narratives for each suicide case were coded to identify the type of IPP involved, a historical presence of IPV, and whether IPP and/or IPV was an immediate precipitating factor of the suicide incident. IPP variables identified IPP that did not indicate violence, such as divorce or marital problems. IPV variables indicated the presence of violence, such as domestic violence or assault. The examples below reflect the type of information available in the incident narrative that can be used to identify presence of IPP and IPV.

Example of IPP with no IPV:

Male (V) hanged himself. He had a history of suicide attempts and had been prescribed medication for depression. The victim had been feeling particularly depressed over an undisclosed physical health problem and relationship problems.

Example of IPP with IPV:

Male (V) shot himself near estranged wife’s residence. He had a history of domestic violence for which she had taken out an EPO against him and was seeking a divorce. After stalking her, he assaulted her and threatened to kill both her and himself. 

To increase confidence in how IPP and IPV suicide circumstances were coded, following the second phase coding, a second coder coded a random sample of all cases (10%) to assess inter-rater reliability. Reviewers agreed on 79.4% of cases in initial independent review. The remaining 20.6% of cases with discrepant codes were discussed in consensus conference and then reconciled. 

The three main reconciled discrepancies were as follows: a) When depression was worded in a way to be considered a precipitating circumstance, the second reviewer coded that as “upset or distraught,” while the first coder did not; b) There were inconsistencies between coders and within coding of individual coders about when to identify “marital problems” when IPP was noted; and c) there were inconsistencies between coders as to when “threat of suicide” and “argument” were enough to code that violence was present. 

Coders discussed how to reconcile and cases with these discrepancies were corrected within the formal coding guidelines and further coding followed these established guidelines. Changes included: 1) defining “upset or distraught” to include depression and anxiety only when it is clearly a precipitating factor of the suicide event and related to IPP or IPV, 2) defining ”marital problems” to include cases involving separation, estrangement, and other IPP variables when the problem is specifically related to the decedent’s legal spouse, and 3) defining “threat of suicide” and “argument” to include cases where the variable identifies verbal or psychological abuse toward their spouse or partner. The variable “threat of suicide” does not include all cases in which the decedent told someone that he or she would end his/her own life, but only cases in which the statement(s) about the suicide was done in a way that was manipulative or threatening. Use of a manipulative suicide threat as a means to control the partner through fear of the threatened suicide is psychological abuse and therefore a form of violence. An example of a manipulative suicide threat can be seen below:

Male (V) shot himself. He was with his estranged wife at the time, of whom he was very jealous. She had left him and the victim told her he would kill himself if he couldn't be with her.

The variable “argument” excludes cases where verbal or psychological abuse is not taking place. Changes to the coding guidelines were completed and raters were in agreement of all coding mechanisms. The data was reassigned as necessary based on coding guideline changes. 

To test our hypothesis, quantitatively, we tested the proportion of cases that have a violence indicator, using OpenEpi (Version 3), an open source calculator. This allowed us to determine if there was a higher than expected number of cases that have a violence indicator when they were identified as having only relationship problems (significant at p=0.05).

## Results

There were a total of 7,008 suicide cases in Kentucky from 2005-2015; circumstance information was collected in cases 5,029 (72%). Where circumstances were known, IPP or IPP and IPV was identified in 1,327 (26%) of these cases. Table 2 shows characteristics of suicides by whether they were IPP-related cases and not IPP-related. Counts of suicide fatalities with IPP, are significantly (χ=15.5, p<0.0001) higher in men compared to women. In cases where alcohol was tested, alcohol was more often cited as related to the suicide when IPP was a factor than when it was not. Although depression was noted much more often in non-IPP related suicide, 19% of decedents were experiencing the combination of IPP and depression. 

**Table 2:** Suicides with no IPPs and IPP –Related Suicides 2005-2015.

NVDRS Variable	Suicides, with no IPP(N=3706)	%	IPP (N=1323***)	%
**Demographic**				
Sex (Male)	2929	79.0	1112	84.1
Race (White)	3527	95.2	1227	92.7
Military Status (Ever Served)	536	14.5	137	10.4
Alcohol (Positive BAC)	678 (2355)	28.8	348 (897)	38.8
Combined (Positive for Any Drug)	1015 (2356)	53.2	330 (893)	37.0
Circumstances**				
Argument	112	3.0	184	13.9
Family Relationship Problem	131	3.5	57	4.3
Financial Problem	294	7.9	143	10.8
Job Problem	316	8.5	142	10.7
Physical Health Problem	996	26.1	112	8.5
Interpersonal Violence-Perpetrator	30	0.8	71	5.4
Mental Health Diagnosis of Depression	1001	27.0	245	18.5
Suicide Attempt History	441	11.9	160	12.1

*These are only cases where circumstances are known. **Circumstances are not mutually exclusive.

The most frequent IPP variables are identified in Table 2 and include: relationship/domestic problems (37%), marital problems (22%), separation/left (15%) and divorce (13%). The most frequent IPV variables, identified in Table 3, include: manipulative suicide threat/intent (24%), argument/fight/altercation (23%, 30% when combined with domestic dispute/argument, which will be used from here forward), assault (not including homicide/suicide (HS) event) (7%) and homicide/suicide combination event (7%). In nearly 32% of cases where IPP was a precipitating circumstance the decedent had made their suicidal ideation known, had disclosed their intent to die by suicide (in a non-manipulative manner), or had previous suicide attempts (data not shown). In several cases the decedent had acted out threatening behaviors (i.e. cutting wrists in front of spouse prior to the suicide, or wielding a knife during an argument and threatening self-harm) and in 17 (1%) cases the decedent had made homicidal threats but then died by suicide (this does not include homicide followed by suicide; data not shown). 

**Table 3:** Intimate Partner Problem (IPP) and intimate partner violence (IPV) indicator presence, counts and percentages, Kentucky, 2005-2015.

Themes	Total IPP/IPV Cases	
Intimate Partner Problems***	(N=1,327)***	%
Relationship/Domestic problem (includes “break-up”)	487	36.7
Marital problems	286	21.6
Separation/left	200	15.1
Divorce	171	12.9
Upset/distraught	83	6.3
Infidelity	78	5.9
Intent to leave	77	5.8
Custody/visitation of children	71	5.4
IPP not specified	58	4.4
Estranged	35	2.6
**Cases with at least one specific Intimate Partner Problem identified**	997+	75.1
Intimate Partner Violence Indicators***		
Manipulative suicide threat/intent	324	24.4
Argument/fight/ altercation	310	23.4
Assault of another person*	96	7.2
Homicide/Suicide Combination	86	6.5
Domestic dispute/argument	85	6.4
IR** stated "violence" or “abusive”	65	4.9
Restraining order (any type)	41	3.1
**Cases with at least one Violence Indicator Identified**	575	43.3

* Not including Homicide/Suicide (H/S) cases. ** IR- refers to the Investigator’s Report ***Categories are not mutually exclusive. +The 997 number is less than the total identified IPP cases (1,327) because investigators could identify IPP on their investiga-tive reports without providing a more detailed narrative, from which we coded type of IPP.

Table 4 shows, that when the IPV variable was available for abstractor selection in 2009 and 2010, intimate partner violence was detected in 10.5% of those cases where there were intimate partner problems; using our coding schema IPV was identified in over 43%. A more specific comparison between Table 3 and 4 indicates that in 2009 and 2010, when KVDRS abstractors had the option of selecting IPV in suicide, over 10% of cases where there were intimate partner problems were also coded as having precipitating IPV, yet in our narrative review of eleven years we found that there was a documented argument/fight/ altercation against their intimate partner in 30% of the cases coded as having precipitating problems, over 7% of the time there was an assault and in 5% there was a history of violence and abuse. 

**Table 4:** Suicides in 2009 and 2010 (Abstractor had the option to select IPP and/or IPV).

Total Suicides (N=719)		%
Suicides with Intimate Partner Problem	181	25.2
Suicides with Intimate Partner Violence	19	2.6
**Suicides with Intimate Partner Problems (N=181)**		%
Suicides with Intimate Partner Violence among those with Intimate Partner Prob-lems	19	10.5

We did reject our null hypothesis that of the cases where IPP was identified there would be 10% of cases with violent indicators as well. We used 10% because that was the percentage found during 2009 and 2010, when IPV was a coding option for suicide, within the NVDRS, so that’s what would be expected for the other years. We found a significant proportion of violent indicators, in years 2005-2015, in cases that were coded as only having IPP (p<0.0000001). 

## Discussion

In Kentucky, for cases of suicide with known circumstances of death, we found the number of male suicides with IPP were significantly higher than female suicides with IPP. While IPP and IPV seem to play similar roles in suicide in men and women, the greater number of men than woman, for whom IPP is a factor, warrants further investigation of this precipitating factor for male suicide. Further research into this intersection of violence and suicide might have the potential to inform effective screening tools referring those at risk to evidence based programs. For example, Kraanen et. al. found that about 50% of patients, with a partner, in substance abuse treatment experienced or perpetrated intimate partner violence. They developed the Jellinek Inventory to identify intimate partner perpetrators and victims. By identifying these co-occurring conditions, substance abuse treatment might be more effective if treatment programs were then modified with consideration of the additional stressor of IPV and in some cases focusing on IPV before substance abuse treatment.^44^

Prevention efforts are dependent on understanding the precipitating factors of suicides, and this requires quality data to recreate an accurate accounting of the suicide event. The NVDRS is the only system where states can gather detailed narratives that provide insight into circumstances. This surveillance system is crucial in understanding population-based risk factors for suicide; if implemented in all 50 states instead of in select states, NVDRS could provide a national view of reasons behind suicide. 

A novel coding mechanism to assess suicide incident narratives was developed. No prior method of identifying type and patterns of violence in IPP related suicide has been published and this has left a void in IPV research. The mechanism developed for this study can easily be replicated to facilitate the identification of IPV in suicide across all NVDRS states. Understanding the risk factors of suicide on a state and national level is imperative for developing prevention efforts. 

Finally, we found our results might be explained within the framework of the interpersonal-psychological theory of suicidal behavior. ^45^ In this theory Joiner proposes that

“an individual will not die by suicide unless s/he has both the desire and the ability…the theory asserts that when people hold two specific psychological states in their minds simultaneously, and when they do so for long enough, they develop the desire for death.” 

The two states are perceived burdensomeness and sense of low belongingness or social alienation. Our results indicate decedents may have developed a fearlessness of pain, injury and death, which aligns with this theory, by experiencing ongoing painful chronic and/or acute stresses, traumas or provocative life experiences; namely relationship discord, rejection and/or violence (37% of cases had relationship/domestic problems). The other part of Joiner’s theory requires the ability to inflict lethal self-injury, which could mean engaging in self-harm behaviors; the more behaviors the more the person will familiarize themselves to weakened fear and increase the risk of a subsequent attempt. Our study showed that the most frequent IPV variable (24%) was manipulative threat/intent and when there were already problems, 32% had made their suicidal ideation known, had disclosed their intent (non-manipulative), or had made actual attempts on their life. In several cases the decedent had acted out threatening behaviors (i.e. cutting wrists in front of spouse prior to the suicide, or wielding a knife during an argument and threatening self-harm) and in 17 (1%) cases the decedent had made homicidal threats but then died by suicide. This is directly aligned with the interpersonal-psychological theory of suicidal behavior. Accidental injury and fighting can exacerbate ongoing exposures to risk of suicide. Our findings show there was an argument/fight/altercation (30%), assault (7%) (not including homicide/suicide (HS) event).^45^

Our study aims were to better understand suicide and IPP as suicides are increasing, and to identify where there was also violence. We reject the null hypothesis in favor of our alternative hypothesis, that there is a significant number of suicide cases involving violence, when only problems have been identified. Because there is no prior method of identifying type and patterns of violence in IPP related suicide, a void has been left in IPV research and the violence lost. Our study brings to light the importance of identifying IPV, in regard to suicide, as those circumstances will often require different prevention approaches.

**Limitations**

This study had several limitations. First, the data were retrospective and relied solely on information completed by CMEs and LLE on investigative death forms. Second, some of the circumstances surrounding the suicide event are potentially subjective. Many are based on the opinion or recollection of family and friends of the decedent or witnesses to the event. Information collected by interviews is subject to recall bias. This bias could be unintentional, but the stigma of suicide and individual motivations of the interviewee could affect their disclosure of information.^8,46^ Third, circumstances were unknown in 30% of suicide cases. Missing values could alter percentages. With increasing technology usage and novel ways of being victimized or perpetrate violence by intimate partners, investigators might assess relationship issues by evaluating on-line personal accounts thereby improving circumstantial understanding and consequently document new trends in cyber abuse.^47^

In approximately 30% of cases with IPP the circumstances were identified as “argument/fight/altercation,” where there was a fight between intimate partners with no unquestionable statement about the perpetrator being the instigator of the argument. However, there is an underlying theme that the partner who perpetrates violence, or acts in a more violent capacity, is generally the person who dies by suicide. Unfortunately, these arguments/fights/altercations often occur in private, prohibiting the ability to determine an accurate understanding of the origins of the argument. 

## Conclusion

Our primary focus of this study was to reduce suicide in Kentucky. Previous studies have found a link between IPP and increased risk of suicide. We did find supporting evidence of our hypothesis that there is a great deal of underlying and outright violence in intimate relationships, which is exacerbating the risk of suicide. We sought to find explanations of why suicides are increasing and what can we do to curb this increase. First, because we did find violence in 43% of cases (significantly rejecting the null hypothesis in favor of our hypothesis) being coded as problems alone, identification of violence in relationships prior to suicide is the first step in a difficult battle to reduce suicide mortality. Prior to this study there was no method of identifying type and patterns of violence in IPP related suicide. This study shows the importance of looking beyond IPPs to see if there is also violence, otherwise we may be missing an entire population of suicides.

Levesque and Chamberland found that overall, young mothers find it difficult to identify themselves as victims of IPV and label their partner’s actions as violent.^48^ Identification of IPV when risk factors, even when victims and perpetrators are in denial, is important in treating co-morbidity as in the example of drug abuse treatment and IPV. With separation and divorce being a triggering event for violence in a relationship, court professionals bear responsibility in identifying when there are IPPs and IPVs and change course accordingly. In fact Beck et. al state, 

“Accurate identification and classification of IPV abuse can also assist clinical researchers designing specialized interventions for couples and individuals experiencing IPV abuse, mental health practitioners who may treat these families, and custody evaluators who may make recommendations to the courts”.^24^

This study presents common characteristics of suicide cases with IPP and IPV from 2005-2015 in Kentucky. It is important to reiterate that common features cannot predict or prove causality. However, we can utilize the identification of existing characteristics of suicide cases to build future studies to determine causal relationships and to inform prevention strategies. Our study is consistent with previous studies as far as relationship problems being a primary risk factor for suicide. Our study added to the existing body of literature in finding that there is missing violence in cases where only problems were identified.

This analysis has resulted in several suggestions for research and practice. Continued surveillance of violent death to assist in identification of risk factors associated with the fatality is key to understanding and correctly categorizing violent deaths. IPV and suicide coding within the NVDRS needs to be evaluated and expanded. For example, IPV should be an available option for coders when the decedent has died by suicide and not only homicide. It is important to, at the least, in all NVDRS states, systematically document if the suicide decedent was a perpetrator of violence/problems or the victim, whether there was a threat of homicide or suicide, if a child witnesses the event and other variables we have identified that are not currently available within the NVDRS IPV module. 

While this study established a novel coding process for incident narratives, this process should be replicated in other states. Replication will allow the external validity of the coding process to be evaluated and allow comparison of states utilizing the same tool to determine whether data follows similar trends or if the results of this study are unique to Kentucky. 

Continual death scene investigation and record keeping improvement is imperative to collect accurate circumstances surrounding suicide events to assist in determining common characteristics. This can be accomplished through training and speaking to investigators, expressing the necessity of accuracy to inform policy and practice. 

**Acknowledgements**

Produced by the Kentucky Injury Prevention and Research Center, a bona fide agent for the Kentucky Department for Public Health. April 2017. Data sources: Kentucky Death Certificate Database, Kentucky Office of Vital Statistics, Cabinet for Health and Family Services; Kentucky Violent Death Reporting System, National Violent Death Reporting System. Data from 2012-2015 are provisional and subject to change.

5U17CE002601-03: Kentucky Violent Death Reporting System from the:

This study was funded by: 

Centers for Disease Control and Prevention (CDC)

Its contents are solely the responsibility of the author and do not necessarily represent the official views of the CDC.
